# Longitudinal relationship between social participation, depressive symptoms, and activity impairment among older patients with arthritis: a moderated mediation analysis

**DOI:** 10.1186/s12877-024-04735-6

**Published:** 2024-02-07

**Authors:** Zeping Yan, Xiaorong Luan, LiJun Meng, Yu Wu, Wenran Qu, Simeng Zhang, Huimin Wei, Shicai Wu

**Affiliations:** 1 School of Nursing and Rehabilitation, Cheeloo College of Medicine, Shandong University, Jinan, China; 2 Beijing Bo’ai Hospital, China Rehabilitation Research Center, Beijing, China; 3 University of Health and Rehabilitation Sciences, Qingdao, China; 4 Qilu Hospital, Shandong University, Jinan, China

**Keywords:** Social participation, Depressive symptoms, Activity impairment, Older, Moderated mediation, Arthritis

## Abstract

**Background:**

Arthritis primarily affects older people and is a prominent cause of their activity impairment. This study aimed to examine the mediating role of depressive symptoms in the relationship between social participation and activity impairment, as well as to determine whether sex moderated the mediating effect.

**Methods:**

A total of 2247 older patients with arthritis were included from the China Health and Retirement Longitudinal Study between 2015 and 2018. We first examined a simple mediation model where depressive symptoms were a mediator between social participation and activity impairment. Furthermore, sex was systematically integrated into the model as a moderator. The mediation model and moderated mediation model were analyzed using PROCESS macro.

**Results:**

Mediation analysis revealed that the association between social participation and activity impairment was partially mediated by depressive symptoms (*B* = -0.10, 95% CI = [-0.14, -0.06]) with intermediary effect of 28.6%. Moderated mediation analysis indicated that mediation model was moderated by sex. The indirect effect of social participation on activity impairment among female patients (*B* = -0.15, 95% CI = [-0.21, -0.09]) was stronger than male patients (*B* = -0.04, 95% CI = [-0.09, -0.01]).

**Conclusion:**

Social participation was the key protective factor associated with depressive symptoms and activity impairment among arthritis patients. Encouraging arthritis patients to social participation and improving the depressive symptoms might avoid activity impairment, especially for female patients.

## Introduction

As a common chronic condition, arthritis is a prominent cause of disability, and thus, is regarded as a critical public health problem [1, 2]. In accordance with a 2017 study on the global burden of diseases, the estimated prevalence of arthritis is accelerating worldwide [3]. Middle-aged and older individuals are the major group suffering from arthritis, and more than 150 million Chinese adults aged ≥ 45 years had physician-diagnosed arthritis in 2014 [1]. Patients tend to experience activity impairment attributed to arthritis [4]. A 23-year follow-up survey established arthritis as an independent and significant predictor of increasing activity impairment [5]. Activity impairment limits the autonomy and causes the dependence of patients with arthritis, typically generating complications and entailing huge economic expenses. Activity limitation is inclined to decrease quality of life and increase the risk of mobility issues, falls, and even death [6]. The detection of modifiable contributing factors related to the activity limitation of patients with arthritis is essential for initiating preventive interventions.

On the basis of the International Classification of Functioning, Disability, and Health framework proposed by the World Health Organization (WHO) and the integrative bio–psycho–social model behind it, activity limitation can be influenced by personal factors (e.g., age and sex), environmental factors (e.g., residence), body functions, and social participation [7, 8]. Activity impairment may result from reduced social participation, which is particularly pronounced among older patients with arthritis [9].

Social participation has been identified as a critical area for action on healthy aging by WHO [10]. A previous report indicated that social participation was link to a greater likelihood of living a disability-free life and encouraging healthy aging [11]. Compared with those without social participation restriction, patients with such restriction reported three to ten times higher prevalence of activity impairment [12]. A prospective cohort study reported that an elderly person who lacked social participation were at a higher risk of experiencing activity impairment after 6 years [13]. However, only a few studies have investigated the mechanism of how social participation influences activity impairment.

Depressive symptoms frequently coexist with activity impairment, and the presence of depressive symptoms is a strong predictor of subsequent activity impairment [14]. A population-based cohort study underscored the importance of depressive symptoms during the onset of activity impairment [15]. Moreover, social participation allows patients to establish social relationships and engage in emotional bonding, exerting a protective effect against depressive symptoms [16]. Accordingly, social participation may predict the incidence of depressive symptoms and future activity impairment, suggesting a potential causal link among the three critical components. Depressive symptoms can be inferred to play a potential mediating role between social participation and activity impairment.

The association among social participation, depressive symptoms, and activity impairment seems to vary by sex. While evidence on the moderating effects of sex in social participation–activity impairment is lacking, specifically among older patients with arthritis, different results have been found in previous studies regarding the overall older population. In particular, some studies have found that social participation exerts stronger effects on depressive symptoms among females than among males [17]. Prior evidence has pointed out that females are more likely to benefit from participating in activities than males [18]; moreover, females generally face a higher risk of depressive symptoms and experience activity impairment compared with males. Thus, sex may be a potential moderator for the mediating effect of depressive symptoms between social participation and activity impairment.

**Fig. 1:**
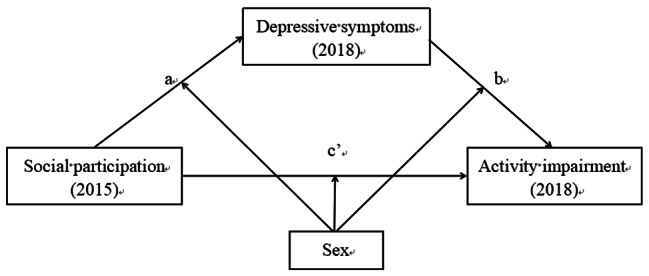
Conceptual framework model

In the present study, we aimed to comprehensively describe the effects of social participation and depressive symptoms on activity impairment in older patients with arthritis. We also intended to analyze the moderating effect of sex. Therefore, this study proposed a moderated mediation model, as shown in Fig. 1. The paths from social participation to depressive symptoms and activity impairment were elucidated using a longitudinal cohort. Our hypotheses were as follows. (1) Depressive symptoms play a mediating role in the relationship between social participation and activity impairment. (2) Sex moderates the mediating effect of depressive symptoms on activity impairment.

## Methods

### Study design and participants

This longitudinal survey analyzed two waves of data (2015 and 2018) from the China Health and Retirement Longitudinal Study (CHARLS). The details of CHARLS were published elsewhere [19, 20]. Briefly, all county-level units in the 28 provinces were stratified in accordance with their regions, urbanization level, and economic development. A total of 150 counties were chosen within each stratum, and 3 rural villages or urban communities were chosen in each county. Moreover, 80 households were chosen from each rural village or urban community via simple random sampling. Every person aged 45 years or older were invited to participate. To fulfill our objective, the inclusion criteria were as follows: physician-diagnosed arthritis, aged 60 years or older in year 2015, and had complete records of the three critical variables in year 2015. The exclusion criteria were as follows: physician-diagnosed cancer or diagnosed with impaired memory function in year 2015. We included an initial sample of 3822 older patients with arthritis in 2015. After excluding 984 patients who did not complete depressive symptoms and 591 patients with missing values of activity impairment in year 2018, 2247 arthritic patients aged ≥ 60 years were retained in the final analysis.

### Measures

#### Social participation

Social participation was assessed via self-reported participation in social activities in year 2015. The participants were interviewed about their engagement in social activities in the past month. We included 11 activities that required yes/no answers: “communicated with friends;” “played mahjong/chess/cards or went to a community club;” “offered free assistance to family, friends, or neighbors who do not live with you;” “went to a sport or social club;” “involved in community-related organizations;” “engaged in volunteer or charity work;” “took care of disabled or sick patients who do not live with you for free;” “attended an educational or training course;” “invested in stocks;” “surfed the Internet;” and “others” if none of the above. Consistent with previous studies [21], the independent variable of social participation was generated as a continuous variable. Each activity that was answered “yes” received 1 point, and total scores ranged from 0 to 11 [22]. Higher scores represented more social participation.

#### Depressive symptoms

Depressive symptoms were assessed via a 10-item version of the Center for Epidemiologic Studies Depression Scale (CES-D) in years 2015 and 2018 [23]. The participants were asked to rate “how often you felt and behaved this way during the past week.” Eight negative items were scored from 0 to 3, and two positive items were reverse coded, obtaining a total score ranging from 0 to 30 [21]. Higher scores indicated greater depressive symptoms. CES-D exhibits good and consistent internal reliability among middle-aged and elderly respondents in China. The Cronbach’s alpha values of CES-D were 0.82 and 0.80 in years 2015 and 2018, demonstrating good reliability.

#### Activity impairment

Activity impairment was assessed through activities of daily living (ADL) and instrumental ADL (IADL) in years 2015 and 2018 [24, 25]. Patients reported whether they experienced difficulties in independently completing six ADL activities (dressing, eating, bathing, getting into/out of bed, toileting, and controlling urination/defecation) and six IADL activities (shopping, cooking, using the telephone, doing housework, managing finances, and taking medications) for more than 3 months. The ADL and IADL scales were coded with scores of 0 to 3 for each item. Continuous scores of activity impairment ranged from 0 to 48, with higher scores indicating poorer activity impairment.

#### Control variables

In accordance with the International Classification of Functioning, Disability, and Health framework, personal factors (age, sex, residence, body mass index, education level, marital status, and self-rated healthy status) and environmental factors (residence) were selected as potential control variables. Linear regression analysis with activity impairment as the outcome variable identified age, sex, marital status, and self-rated healthy status as independent predictors, which were included in the moderated mediation model.

### Ethical consideration

The CHARLS survey was approved by the institutional review board of Peking University (IRB00001052-11015), and all patients offered informed consent voluntarily [26].

### Statistical analysis

Data analyses were performed using SPSS version 26.0, with PROCESS macros created by Hayes [27]. Sample characteristics were described using frequency (percentage). The three key variables were displayed as the mean (standard deviation), and Spearman correlation analysis was conducted to test the correlations among these variables. Considering our hypotheses, the mediation model for confirming whether depressive symptoms mediated the relationship between social participation and activity impairment was tested using Model 4 in PROCESS, calculated using 5000 bootstrap procedures. The mediating effect was significant if the 95% confidence interval (CI) of the indirect effect (a*b) did not cross 0. Then, the moderated mediation models were estimated by Models 59 and 58 by using a bootstrapping approach. Zero was located within the 95% CI, indicating that the effect was insignificant. Given that age, marital status, self-rated health, and the three key variables were all related, these variables were controlled in all the models. In addition, depressive symptoms and activity impairment in year 2015 served as covariance in the model. All the tests were two-tailed, and *p* < 0.05 was considered statistically significant.

## Results

### Study sample

The characteristics of the participants are listed in Table 1. Mean age was 66.98 years (SD = 5.53) at baseline. Among the 2247 patients with arthritis, 958 (42.6%) were male and 1289 (57.4%) were female. The majority of the patients lived in rural areas (76.1%) and married (81.4%). Only a small fraction of older patients with arthritis self-rated their health as good (12.2%).

**Table 1:** Characteristics of the participants and correlation coefficient among the variables (*N* = 2247)

Variables	M ± SD/n (%)	SP(T1)	DS(T1)	AI (T1)	DS (T2)
Age	66.98 ± 5.53				
Sex					
Male	958 (42.6)				
Female	1289 (57.4)				
BMI					
<18.5	121 (6.2)				
18.5 ~ 24.9	1184 (60.5)				
≥25	653 (33.4)				
Education level					
Primary school or below	974 (76.5)				
Junior high school	220 (17.3)				
High school or above	80 (6.3)				
Residence					
Urban	536 (23.9)				
Rural	1711 (76.1)				
Marital status					
Married	1830 (81.4)				
Unmarried/widowed/divorced	417 (18.6)				
Self-rated Health					
Good	274 (12.2)				
Fair	1157 (51.5)				
Poor	814 (36.2)				
Social Participation (T1)	0.81 (1.00)	-			
Depressive Symptoms (T1)	10.08 (6.85)	-0.110^***^	-		
Activity Impairment (T1)	10.38 (6.96)	-0.089^***^	0.352^***^	-	
Depressive Symptoms (T2)	2.56 (4.04)	-0.147^***^	0.523^***^	0.312^***^	-
Activity Impairment (T2)	2.66 (4.26)	-0.128^***^	0.310^***^	0.480^***^	0.378^***^

M: Mean; SD: standard deviation; DS: depressive symptoms; AI: Activity impairment; ^***^p < 0.001; T1: Time 1:2015; T2: Time 2:2018

### Correlations among study variables

The results of the bivariate correlations among the study variables are presented in Table 1. Social participation in year 2015 was negatively correlated with depressive symptoms (*r* = − 0.147, *p* < 0.001) and activity impairment (*r* = − 0.128, *p* < 0.001) in year 2018, indicating that better social participation was associated with a lower degree of depressive symptoms and activity impairment. Depressive symptoms were positively correlated with activity impairment (*r* = 0.378, *p* < 0.001) in year 2018, suggesting an association between poorer depressive symptoms and a higher degree of activity impairment.

### Mediation analysis

Table 2 presents the results of the mediation analysis. First, social participation was negatively associated with activity impairment (Path c), with a significant unstandardized regression coefficient (*B* = − 0.35, *p* < 0.001). Second, the direct effects of social participation on depressive symptoms (Path a, *B* = − 0.70), depressive symptoms on activity impairment (Path b, *B* = 0.14), and social participation on activity impairment (Path cʹ, *B* = − 0.25) were significant (all *p* < 0.001). Moreover, a bootstrapping analysis revealed that social participation was partially correlated with activity impairment through depressive symptoms (*B* = − 0.10, 95% CI = [− 0.14, − 0.06]). Taken together, depressive symptoms partially mediated the relationship between social participation and activity impairment, accounting for 28.6% of the total effect.

**Table 2:** The results of the mediating analysis

Model summary	*B*	*SE*	*t*	*p*	95% CI	
Total effect						
Path c, X predicting Y	-0.35	0.07	-4.67	< 0.001	[-0.49, -0.20]	
Direct effect						
Path a, X predicting M	-0.70	0.12	-5.68	< 0.001	[-0.94, -0.46]	
Path b, M predicting Y	0.14	0.01	11.26	< 0.001	[0.12, 0.16]	
Path c’, X predicting Y	-0.25	0.07	-3.42	< 0.001	[-0.39, -0.11]	
Indirect effect of X on Y through M						
Unstandardized	-0.10	0.02			[-0.14, -0.06]	

X: Independent variable (Social participation); M: Mediator (Depressive symptoms); Y: Dependent variable (Activity impairment)

### Moderated mediation analysis

**Fig. 2:**
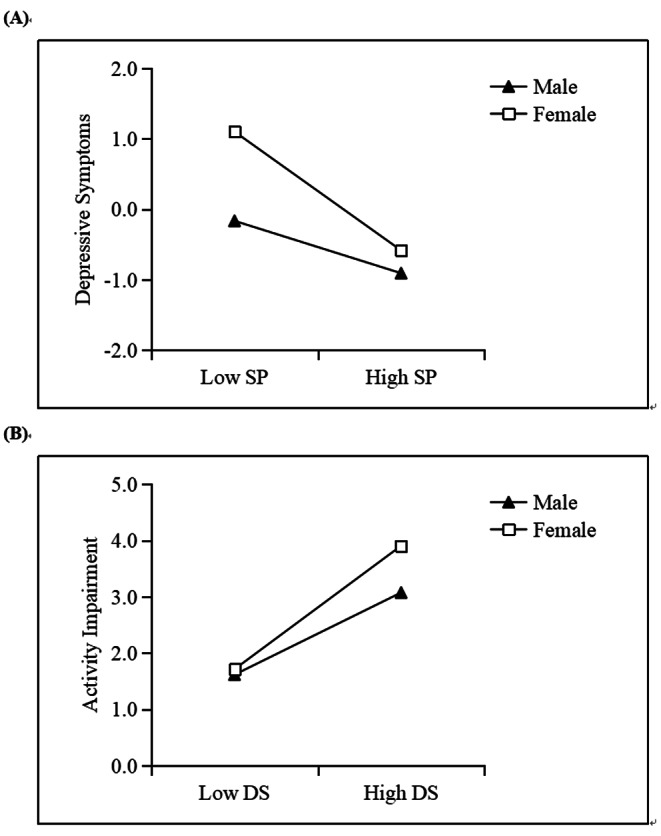
Test of moderation model in which the relationship between social participation and activity impairment mediated by depressive symptoms is moderated by sex. SP: social participation; DS: depressive symptoms

Table 3; Fig. 2 outline the results of the moderated mediation analysis. In Model 59, the interaction term between social participation and sex was significantly related to depressive symptoms (*B* = − 0.52, *p* < 0.05), and the interaction term between depressive symptoms and sex was significantly related to activity impairment (*B* = − 0.05, *p* < 0.05). However, the interaction term between social participation and sex was not significantly associated with activity impairment (*B* = − 0.04, *p* > 0.05), indicating that sex did not moderate the relationship between social participation and activity impairment.

**Table 3:** Results of the moderated mediation analysis

Variables	Model 59				Model 58			
	Depressive symptoms (T2)		Activity impairment (T2)		Depressive symptoms (T2)		Activity impairment (T2)	
	*B*	*t*	*B*	*t*	*B*	*t*	*B*	*t*
Age	-0.04	1.70	0.05	3.97^***^	-0.04	1.70	0.05	3.97^***^
Marital status	0.78	2.39^*^	0.17	0.90	0.78	2.39^*^	0.17	0.89
Self-rated Health	0.49	3.56^***^	0.32	4.01^***^	0.49	3.56^***^	0.32	4.02^***^
Depressive symptoms (T1)	0.13	3.90^***^	-0.02	-1.67	0.13	3.90^***^	-0.02	-1.67
Activity impairment (T1)	0.46	23.09^***^	0.47	23.60^***^	0.46	23.09^***^	0.47	23.62^***^
Sex	0.85	3.33^***^	0.46	3.10^**^	0.85	3.33^***^	0.46	3.09^**^
Social participation (T1)	-0.41	-2.26^*^	-0.22	-2.04^*^	-0.41	-2.26^*^	-0.24	-3.31^**^
Social participation (T1) × Sex	-0.52	-2.13^*^	-0.04	-0.29	-0.52	-2.13^*^	-	-
Depressive symptoms (T2)	-	-	0.11	5.75^***^	-	-	0.10	5.75^***^
Depressive symptoms (T2) × Sex	-	-	0.05	2.37^*^	-	-	0.05	2.44^*^
R^2^	0.32		0.37		0.32		0.181	
F	128.93^***^		128.65^***^		128.93^***^		133.882^***^	

^*^p < 0.05; ^**^p < 0.01; ^***^p < 0.001; T1: Time 1:2015; T2: Time 2:2018

Model 58 was used to test the moderation mediation effect further, and the index of moderated mediation was significant (*B* = − 0.10, 95% CI = [− 0.17, − 0.04]), indicating that the indirect effect of social participation on activity impairment was moderated by sex. For male patients, the indirect effect of social participation on activity impairment was significant (*B* = − 0.04, 95% CI = [− 0.09, − 0.01]). For female patients, the indirect effect on activity impairment was stronger (*B* = − 0.15, 95% CI = [− 0.21, − 0.09]). Simple slope tests showed that the association between social participation and depressive symptoms was stronger for female patients (*B*_*simple*_ = − 0.93, *p* < 0.001) than for male patients (*B*_*simple*_ = − 0.41, *p* < 0.05) (Fig. 2A). In addition, the association between depressive symptoms and activity impairment was stronger for female patients (*B*_*simple*_ = 0.11, *p* < 0.001) than for male patients (*B*_*simple*_ = 0.16, *p* < 0.05) (Fig. 2B).

## Discussion

By using the national representative sample of older patients with arthritis from CHARLS, this study acquired a more nuanced understanding of the relationship between social participation and activity impairment. The results indicated that depressive symptoms played a mediating role between social participation and activity impairment, supporting the first hypothesis. Furthermore, this study found that sex moderated the first and second halves of the mediating effect of depressive symptoms between social participation and activity impairment. Female patients with lower social participation and more depressive symptoms exhibited poorer activity impairment than male patients, supporting the second hypothesis.

Our results are aligned with those of the previous literature, demonstrating that patients who actively participate in social activities, including social interactions and leisure activities, experience less activity impairment [11]. Active social participation is considered a crucial component of positive aging [28], providing opportunities for patients to access material resources of health-relevant information and acquire adequate social support. Several studies have demonstrated that social participation considerably influences mental health disorders and activity impairment [9, 16, 29, 30]. Social participation encourages patients to practice activities, and patients with arthritis are advised to engage in social participation to reduce the risk of activity impairment.

In the search for underlying mechanisms, our results suggested that the relationship between social participation and activity impairment was partly explained by depressive symptoms, as hypothesized. Social participation allows patients to develop social roles and relationships, which can improve depressive symptoms, preventing activity impairment [31]. Our findings also filled the knowledge gap by examining the pathway in which social participation may buffer depressive symptoms, avoiding the development of activity impairment. Intervening only to increase social participation is not normally extremely effective in improving activity impairment [32]. Clinicians should consider the mediating role of depressive symptoms, and depressive symptoms can be enhanced through interventions, such as emotion regulation, meditation, and music therapy, to protect against activity impairment [33]. While encouraging and helping older patients with arthritis to engage actively in social participation, healthcare professionals should also monitor any signs of depressive symptoms and modify medication dosages for arthritis and depression. They should also be mindful of drug interactions.

As expected, sex moderated Path a (social participation–depressive symptoms) and Path b (depressive symptoms–activity impairment) in the mediation model. In particular, the links of Paths a and path b were more salient for females than males. Females practiced participation less frequently in their leisure time and reported more depressive symptoms, highlighting the need for sex-specific interventions with this subsample. The noxious effects of depressive symptoms on activity impairment were aggravated with sex because female patients may lack social support to cope with depressive symptoms [34]. This finding is an indication that interventions that aim to alleviate activity impairment by coping with depressive symptoms may be well suited for female patients with arthritis.

### Clinical implications

Taken together, our research findings have essential implications for the prevention of activity impairment among older patients with arthritis. Social participation helps prevent depressive symptoms and activity impairment, and paramedics should give particular attention to encourage older patients with arthritis to engage in social participation. Considering that female arthritic patients face a higher risk of activity impairment than male patients, intervention programs and policy initiatives should also give female patients more attention. The government and community are expected to improve sites and recreation facilities, build formal volunteer organizations, and optimize access to healthcare services, facilitating the social participation of patients with arthritis. Meanwhile, community health nurses should consider the psychological health of patients with arthritis, particularly females, and assist them in expressing their feelings and receiving support from their family and others [35].

### Limitations and future research directions

Some caveats exist in interpreting our results. First, physician-diagnosed arthritis was self-reported. However, this case definition was sensitive in identifying arthritis. Second, the CES-D scale was also a self-report of symptoms. Hence, social desirability bias may have prevented patients from providing completely truthful responses. Consequently, a more impartial review technique is recommended to be applied in the future. Third, other factors which may influence activity impairment, such as physical activity level and whether patients take antidepressants, were not included in this study. Fourth, bidirectional relationships may exist between depressive symptoms and activity impairment, and future studies can elucidate these relationships by using multiple-wave longitudinal data. Lastly, all the patients included in this study had a common cultural background. Therefore, we recommend that further research be conducted in various cultural contexts to verify the stability of the relationship.

## Conclusions

We highlighted the psychosocial mechanisms of preventing and improving activity impairment among older patients with arthritis. The mediating role of depressive symptoms between social participation and activity impairment and the moderating effect of sex between depressive symptoms and activity impairment were identified. The encouragement of arthritic patients to participate actively in social activities and the timely detection and management of depressive symptoms are crucial for preventing activity impairment, particularly for female patients with arthritis.

## Data Availability

The CHARLS datasets are publicly available at the Peking University (http://charls.pku.edu.cn/en) and can be obtained after submitting a data use agreement to the CHARLS team.

## Abbreviations

CHARLS: The China Health and Retirement Longitudinal Study
CES-D: Epidemiologic Studies Depression Scale
ADL: Activities of Daily Living
IADL: Instrumental activities of daily living
CI: Confidence Interval
M: Mean
SD: Standard Deviation
DS: Depressive Symptoms
AI: Activity Impairment
SP: Social Participation
T1: Time 1:2015
T2: Time 2:2018
